# Time at home during the COVID-19 pandemic: a prospective examination of psychosocial health in people with and without type 2 diabetes using digital phenotyping

**DOI:** 10.1093/eurpub/ckac129.703

**Published:** 2022-10-25

**Authors:** A McInerney, N Schmitz, M Matthews, S Deschenes

**Affiliations:** 1 University College Dublin, Psychology, Dublin, Ireland; 2 Tuebingen University, Population-Based Medicine, Tuebingen, Germany; 3 McGill University, Psychiatry & Epidemiology, Montreal, Canada

## Abstract

**Introduction:**

Societal restrictions due to COVID-19 have had a profound effect on our ability to connect with one another and limited our personal mobility. There is evidence that loneliness, social isolation, and psychological distress increased during restrictions for people with diabetes. Fluctuating restrictions provide a unique opportunity to utilise continuous GPS data from personal smartphones (digital phenotypes) to explore the relationship between time-at-home and psychosocial health for people with diabetes. This study aims to (1) describe the digital phenotypes of time-at-home during varying societal COVID-19 restrictions for people with and without type 2 diabetes and (2) to explore associations between these digital phenotypes and loneliness, social support, and other psychosocial factors and compare for people with and without type 2 diabetes.

**Methods:**

Data come from a longitudinal observational study in the Republic of Ireland that ran between March and August 2021. Participants are seventy-four adults (64.8% female; median age-group = 50-54) with (N = 40) and without (N = 34) diabetes. Continuous GPS data were recorded for 2 months through the Beiwe smartphone application. Loneliness (UCLA-3), social support (MSPSS), diabetes stigma (DSAS-2; diabetes cohort only) as well as other demographic, psychosocial, and lifestyle questionnaires were assessed at baseline, 1 month, and 2 months follow-up.

**Analysis:**

GPS data are being processed. The GPS-derived features of time-at-home, overall movement, and location variance will be computed. Associations between these digital phenotypes and psychosocial factors will be explored and changes over time examined using multilevel modeling.

**Conclusions:**

We expect this study to be the first to describe and compare the digital phenotypes of people with and without diabetes during varying societal COVID-19 restrictions, providing new insights into the effects of such policies on the psychosocial health of people with diabetes.

